# Decision-Making Dilemma in Preoperative Cardiac Evaluation: Should We Turn the Page or Close the Book?

**DOI:** 10.7759/cureus.21151

**Published:** 2022-01-12

**Authors:** Shikha Jha

**Affiliations:** 1 Internal Medicine, Saint Peter’s University Hospital, New Brunswick, USA

**Keywords:** coronary artery bypass graft surgery, coronary artery angiogram, exercise stress test, multivessel coronary artery disease (mvcad), disease risk assessment, pre operative, cardiac clearance

## Abstract

Coronary artery disease is one of the most dreadful and life-threatening diseases out of all cardiac diseases. The diagnosis and management of coronary artery disease comprise stepwise approaches. All these approaches are mostly guideline-driven. While the majority of the time, guidelines help us take the most appropriate care, exceptions do exist. For example, patients may have unusual risk factors and abnormal test results; however, they do not fit into the guideline algorithm to proceed further.

This case report of a 68-year-old male patient depicts a true example of such a situation. He presented to the cardiologist's office for pre-operative cardiac evaluation for urological surgery. In view of associated risk factors, an exercise stress test was done, which showed critical abnormalities. As per the pre-operative cardiac assessment guidelines, the patient did not meet the criteria for further testing. However, a clinician's strong judgment and persistent negotiation superseded those barriers. Given critical abnormalities of the exercise stress test, the patient underwent cardiac catheterization. He was found to have triple vessel disease on cardiac catheterization. The scheduled surgery was withheld, and the patient underwent a coronary artery bypass graft. This life-threatening condition could have been easily missed if only the guidelines were to be followed. While guidelines cover a significant portion of the bell curve, this case report represents the importance of not missing the tail ends of the curve. It enhances the importance of thinking out of the box based on clinical training and expertise.

## Introduction

Cardiovascular disease is one of the foremost causes of death in the United States [1]. Coronary artery disease is the most common type of heart disease. The annual number of deaths due to heart disease is about 659,000, that is, one in every four deaths [2]. Heart attacks account for a significant portion of these deaths. About one in five of these heart attacks are silent, where the person is not aware of the heart damage [3]. Patients with significant coronary artery disease can be asymptomatic with a normal physical exam. In such scenarios, there are high chances of missing the diagnosis. Due to the complexity of coronary artery diseases, there do exist diagnostic challenges related to it. Different available tests, including ECG, exercise stress test, myocardial perfusion scan, and coronary angiogram, have a variable amount of sensitivity, specificity, and positive and negative predictive values. The stepwise diagnostic approach for pre-operative cardiac assessment has been designed to incorporate the clinical picture and appropriate choice of these tests. However, there can be situations where a clinician's judgment to proceed with a diagnostic test for a patient may not fit in with these ideal criteria. Hence, the dilemma of decision-making can be challenging for a physician in those situations for patient care.

## Case presentation

**Figure 1:**
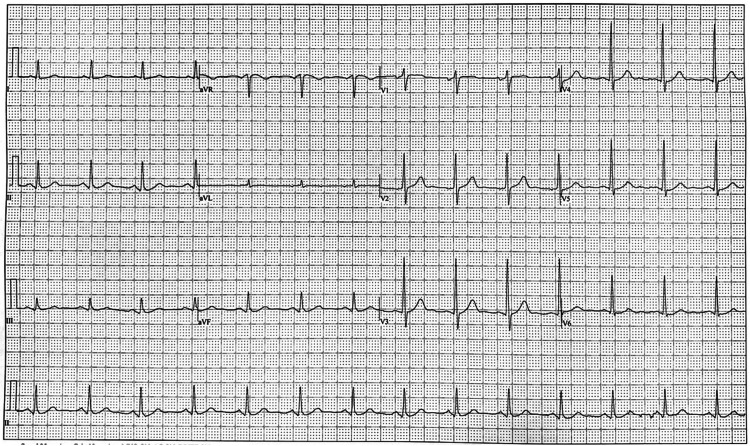
ECG showing normal sinus rhythm.

The patient is a 68-year-old male who came to the cardiologist's office for a pre-operative cardiac evaluation. He stated that he had been scheduled for prostatectomy surgery the same month by his urologist. On the day of his visit, he did not have active cardiorespiratory complaints, such as chest pain, shortness of breath, palpitations, dizziness, and syncope episodes. His medical history had been significant for hyperlipidemia, hypertension, prostate cancer, and type 2 diabetes. His medications included aspirin, telmisartan, atorvastatin, and metformin. Family history was pertinent for hypertension, diabetes, and death due to cardiac causes in parents. On the day of his visit, his vitals and physical exam were within normal limits. The metabolic equivalents (METs) score was 8 points, and the revised cardiac risk index (RCRI) score was class 1 risk, which is 3.9% 30-day risk of death, myocardial infarction, or cardiac arrest. The ECG showed normal sinus rhythm, with no acute changes (Figure 1).

**Figure 2:**
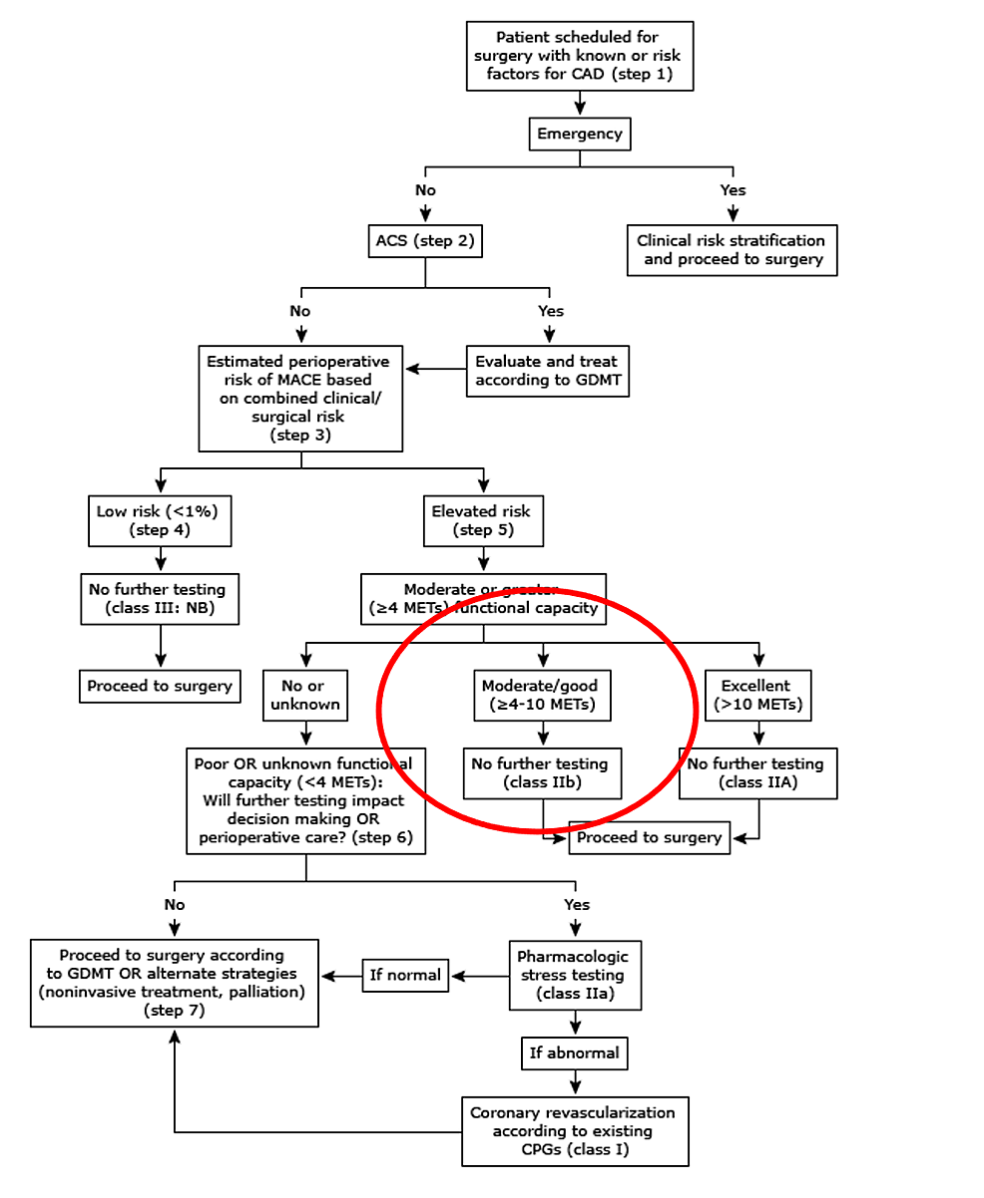
2014 ACC/AHA guideline on perioperative cardiovascular evaluation and management of patients undergoing noncardiac surgery. ACC: American College of Cardiology; AHA: American Heart Association.

All the lab works, including the complete blood count, basic metabolic profile, lipid profile, and glycosylated hemoglobin (HbA1c), were within normal limits. Therefore, as per the stepwise approach to perioperative cardiac assessment guidelines (Figure 2) [4], the patient qualified for proceeding with the surgery with no further tests needed.

**Figure 3:**
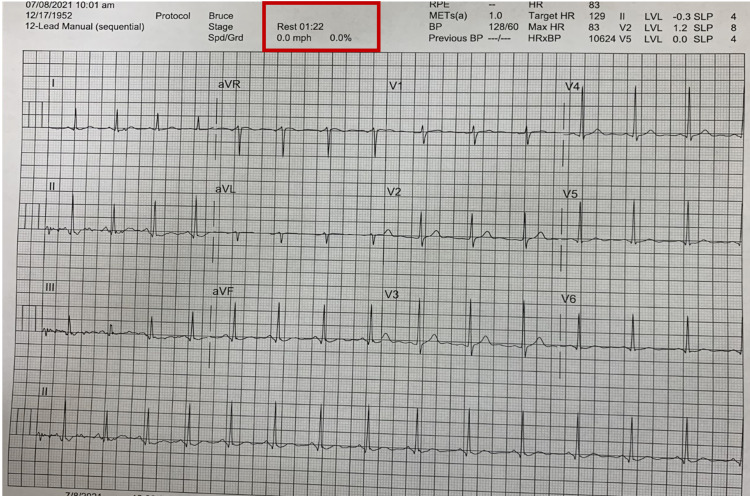
Exercise ECG at 01:22 minutes.

The diagnostic dilemma at this point was whether the patient should be allowed to proceed with the surgery or should further evaluation be done. The decision was made to get an exercise stress test done for the patient because of strong risk factors, including his ethnicity, several chronic medical conditions, and strong family history. The exercise stress test was done based on Bruce protocol. As per the exercise stress test ECG, no significant changes were noted at 01:22 minutes (Figure 3).

**Figure 4:**
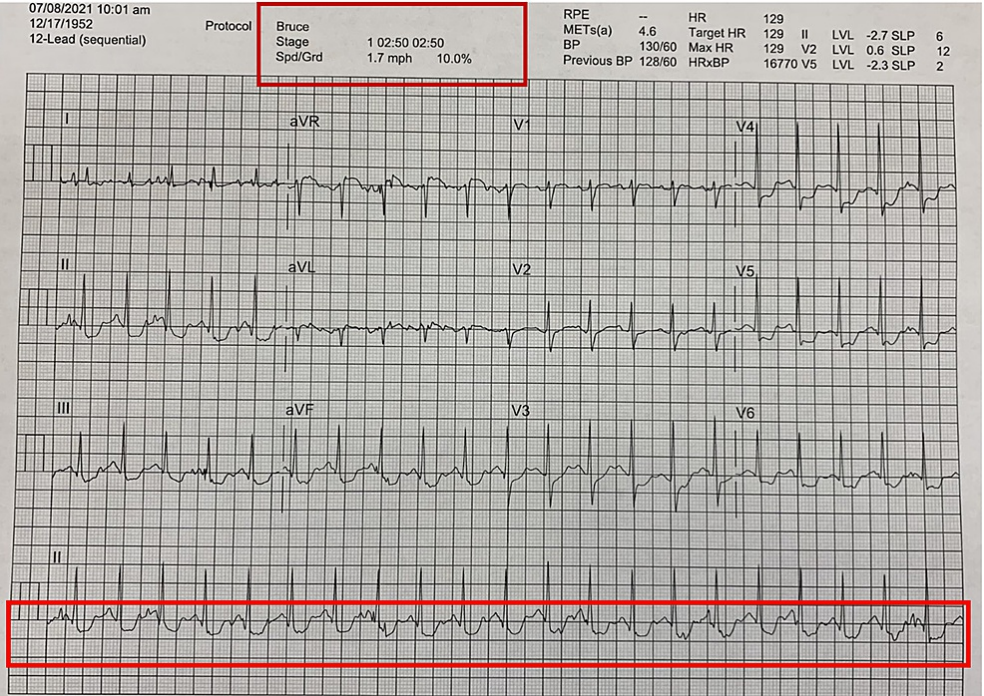
Exercise ECG at 02:50 minutes.

At 02:50 minutes, significant ST depressions were noted in multiple leads (Figure 4).

**Figure 5:**
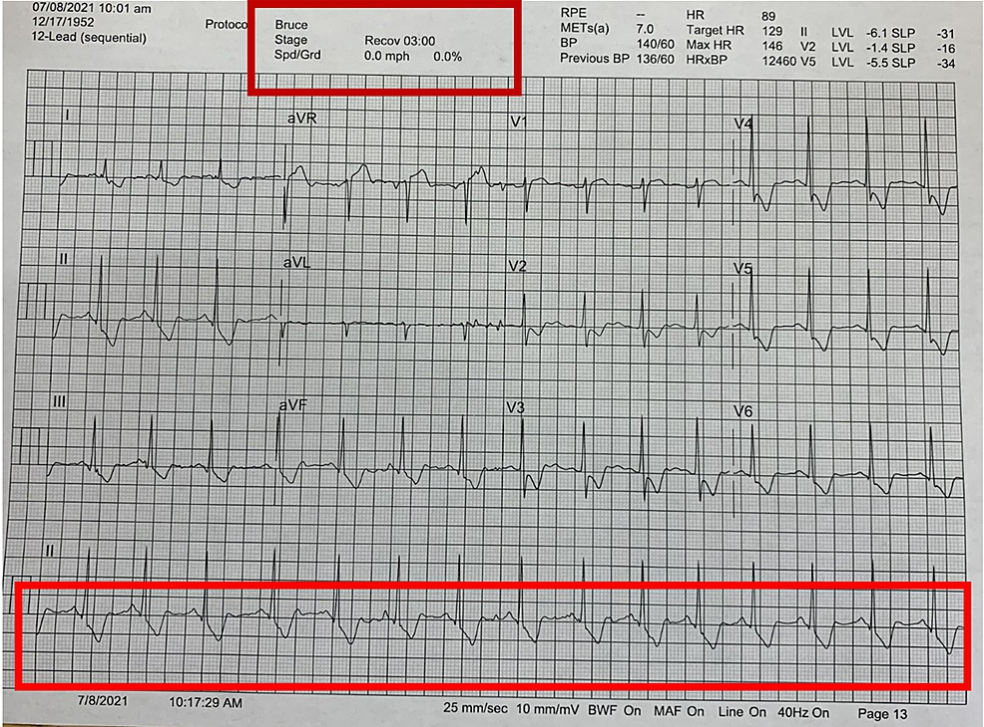
Exercise ECG in the recovery phase (at 03:00 minutes).

The concerning aspect was persistent ST depression in most leads and ST elevations in some of the leads even during the recovery phases at 03:00 minutes (Figure 5).

**Figure 6:**
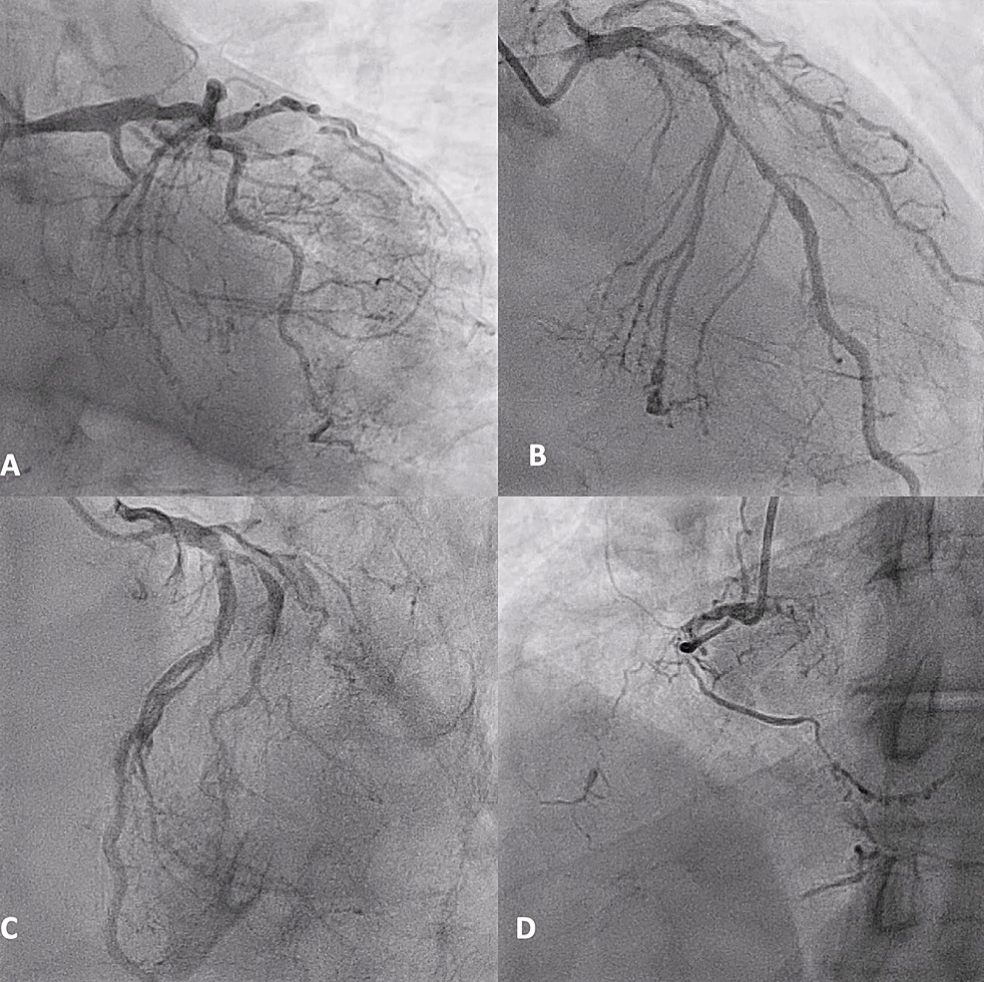
Coronary angiogram showing multi-vessel occlusive disease.

The critical abnormalities on the ECG raised strong suspicion for underlying coronary artery disease. The diagnostic dilemma at this stage was whether cardiac clearance should be given or not. The options were to proceed with the surgery without further intervention or proceed with medical management such as beta-blockers or hold the surgery and perform further investigation, such as myocardial perfusion scan or cardiac catheterization. The cardiac troponins checked were within normal limits. Unfortunately, the patient did not meet the appropriate criteria for further investigation based on the algorithm. The decision was made to perform cardiac catheterization in view of the highest diagnostic accuracy of this test. A lot of resistance was encountered initially from the patient's insurance due to criteria not being met for further tests. However, on persistent discussion with the insurance, approval was obtained for cardiac catheterization. The patient was well explained about the abnormal exercise stress test results and the importance of ruling out severe coronary artery disease before proceeding with the surgery. The patient agreed to the plan. The cardiac catheterization was done (Figure 6), and he was found to have the multivessel disease (Figure 6A-6D).

The prostatectomy surgery was withheld at that time, and the patient underwent a coronary artery bypass graft in view of his multivessel occlusive disease.

## Discussion

All the patients planning for non-cardiac surgery should undergo a risk assessment of cardiac events perioperatively. This assessment aims to help the clinician and the patient analyze the risks and benefits of the surgery. The clinical scenario of this case had a clear objective of the pre-operative cardiac assessment. Patients with several chronic medical conditions, including diabetes, hypertension, and hyperlipidemia, require cardiac optimization prior to elective surgery. Several validated models are used based on the history obtained, physical exam, ECG, and type of surgery. The most common scores used are the METs and RCRI scores. The METs score assesses the functional capacity of the patient [5]. The RCRI score assesses the risk of major cardiac complications such as nonfatal myocardial infarction, cardiac deaths, cardiogenic pulmonary edema, complete heart block, and nonfatal cardiac arrest [6]. Therefore, as per the American College of Cardiology/American Heart Association guideline on perioperative cardiovascular evaluation, our patient qualified for proceeding with the surgery without further investigation needed. The first dilemma in the decision-making occurred at the initial risk evaluation, where everything looked perfect based on the checklist. It was an easy step to go ahead and give clearance at that point. However, the clinician's judgment did not allow the closure of the book at this point. Several predisposing risk factors of the patient brought concerns to his outcome. The goal was to turn on to the next page of investigation to rule out the serious cardiac risk confidently. Hence, an exercise stress test was obtained. This stress test was obtained based on the Bruce protocol, which is divided into successive three-minute stages [7]. The most pertinent change on the ECG during exercise is the alteration in the ST segment. The ST-segment depression during exercise is one of the most common signs of myocardial ischemia. The stress test is considered abnormal when there is more than or equal to 1 millimeter (mm) of down-sloping or horizontal depression in one or more leads. Our patient, in this case, had significant ST depressions during the exercise and the recovery phase. These findings were very concerning for significant coronary artery disease.

The second dilemma of decision-making occurred at this point. The options were to let the patient proceed with the surgery without further intervention, proceed with surgery with medical management such as beta-blockers [8], or withhold the surgery until further investigations such as myocardial perfusion scan or cardiac catheterization. Based on the abnormal exercise stress test, the decision was made to hold the surgery until further investigation is done to rule out the significant coronary artery disease. 

Based on the diagnostic accuracy of the test, patients with indirect screening results positive for coronary artery disease may or may not have an actual disease. In case of intermediate or high-risk findings on the screening result, subsequent testing should be considered to rule out myocardial ischemia. Though radionuclide myocardial perfusion scan has higher sensitivity and specificity than exercise ECG, there is still a high chance of missing out on the actual abnormality. For instance, a meta-analysis performed to compare the test results in intermediate pretest coronary artery disease patients showed that myocardial perfusion scan has around 70-80% sensitivity and around 70% specificity in most patients [9]. Due to limitations on the diagnostic accuracy of the noninvasive tests, as mentioned, the dilemma was whether choosing an invasive test such as coronary angiography was more beneficial than simply doing a noninvasive test. Though the effectiveness of coronary revascularization for the treatment of silent coronary artery disease is limited by data evidence, there are several studies available that suggest that coronary angiography followed by revascularization significantly improves outcomes in these patients [10]. The patient was well explained about his abnormal test discussed earlier and the importance of ruling out heart disease.

The next challenge was to negotiate and persuade the insurance company about the same. The patient eventually underwent cardiac catheterization, which showed significant occlusion in multiple coronary vessels, a life-threatening condition. The final decision-making dilemma was whether to opt for coronary artery bypass graft or percutaneous coronary intervention with a drug-eluting stent. There have been several studies stating the merits and demerits of both coronary artery bypass graft and percutaneous coronary intervention with a drug-eluting stent. In terms of multivessel disease, coronary artery bypass graft remains the preferred choice of revascularization due to reduced risk of mortality and repeat revascularization [11].

## Conclusions

In conclusion, this case report essentially lays out several challenging aspects encountered during pre-operative cardiac evaluation decision-making. Biases and limitations do exist in guidelines and algorithms. Therefore, strong clinical judgment should be our utmost priority during patient care. The diagnostic accuracy of several tests has its own limitations. Hence, individualizing the test choice based on a particular patient case should be the step. Despite all the odds, the ultimate goal should be to catch a near-miss. The dilemma of decision-making at several steps will challenge us to either turn the page to take the next step or close the book without further intervention. There is no denying that there are always pros and cons associated with each decisive step. However, the morale should be to trust one's clinical skills and amalgamate them with a wise diagnostic approach for best patient care.
